# Selective RNAi-silencing of Schwann cell Piezo1 alleviates mechanical hypersensitization following peripheral nerve injury

**DOI:** 10.21203/rs.3.rs-3405016/v1

**Published:** 2023-10-16

**Authors:** Brandon Itson-Zoske, Uarda Gani, Alexander Mikesell, Chengsheng Qiu, Fan Fan, Cheryl Stucky, Quinn Hogan, Seung Min Shin, Hongwei Yu

**Affiliations:** Medical College of Wisconsin; Medical College of Wisconsin; Medical College of Wisconsin; Medical College of Wisconsin; Medical College of Georgia; Medical College of Wisconsin; Medical College of Wiconsin; Medical College of Wisconsin; Medical College of Wisconsin

**Keywords:** Piezo1, Peripheral nervous system, Schwann cells, AAVolig001, Neuropathic pain, Sciatic nerve injection

## Abstract

We previously reported functional Piezo1 expression in Schwann cells of the peripheral nervous system. This study is designed to further investigate the role of Schwann cell Piezo1 in peripheral nociception. We first developed an adeno-associated viral (AAV) vector that has primary Schwann cell tropism after delivery into the sciatic nerve. This was achieved by packing AAV-GFP transcribed by a hybrid CMV enhancer/chicken β-actin (CBA) promoter using a capsid AAVolig001 to generate AAVolig001-CBA-GFP. Five weeks after intrasciatic injection of AAVolig001-CBA-GFP in naïve rats, GFP expression was detected selectively in the Schwann cells of the sciatic nerve. A short hairpin RNA against rat Piezo1 (PZ1shRNA) was designed that showed efficient physical and functional knockdown of Piezo1 in NG108 neuronal cells. A dual promoter and bidirectional AAV encoding a U6-driven PZ1shRNA and CBA-transcribed GFP was packed with capsid olig001 (AAVolig001-PZ1shRNA), and AAV was injected into unilateral sciatic nerve immediately after induction of common peroneal nerve injury (CPNI). Results showed that the development of mechanical hypersensitivity in the CPNI rats injected with AAVolig001-PZ1shRNA was mitigated, compared to rats subjected with AAVolig001-scramble. Selective *in vivo* Schwann cell transduction and functional block of Piezo1 channel activity of primary cultured Schwann cells was confirmed. Together, our data demonstrate that 1) AAVolig001 has unique and selective primary tropism to Schwann cells via intrasciatic delivery and 2) Schwann cell Piezo1 contributes to mechanical hypersensitivity following nerve injury.

## Introduction

Schwann cells (SCs), the specialized glial cells of the peripheral nervous system (PNS), are critical for the function and health of the PNS. Although exploration of the pathogenesis underlying neuropathic pain has centered on neuronal mechanisms, accumulating evidence suggests that SCs, which are physiologically exposed to mechanical stresses and detect nerve injury, play important roles in the development and maintenance of neuropathic pain.^1–6^

Piezo channels, including Piezo1 and Piezo2, are mechanosensitive channels that are expressed widely throughout the body of mammals.^7,8^ Sensory neuronal Piezo2 contributes to peripheral nociception,^9^ while sensory neuron-resided Piezo1 is reported to transduce mechanical itch but not nociception.^10^ Seminal studies propose that Piezo1 is a polymodal sensor of diverse mechanical forces primarily expressed in non-sensory tissues, playing essential roles in a wide range of physiological processes in multiple organs and systems ^11^ and likely participates in other biological functions yet to be discovered. Recent studies found enriched Piezo1 (and Piezo2) expression in the SCs.^12^ SCs have been shown to be required for mechanosensation,^13^ suggesting that Piezo1 may participate in the mechanosensation of SCs. Using a classic Cre-Loxp strategy via a myelin protein zero (PMZ) promoter transcribing Cre for a conditional Piezo1 or 2 ablations in myelinating SCs (mSCs), a study shows that Piezo1 inhibits myelination while Piezo2 facilitates myelin formation in the PNS.^12^ We reported that Piezo1 is enriched in rat SCs, including mSCs and non-myelinating SCs (nmSCs). SCs-Piezo1 is sensitive, compared to sensory neurons and other non-neuronal cells in the PNS, to Piezo1 agonist Yoda1 stimulation that induces mechanical hypersensitivity when applied into sciatic nerve in rats.^14^ This suggests that SCs-Piezo1 activation may contribute to the enhanced mechanical nociception.

This study aims to investigate whether AAV-mediated RNA interference (RNAi) gene silencing approach can be utilized to study the role of SCs-Piezo1 in peripheral nerves. We characterized the tropism and transduction efficiency of a novel ‘oligotropic’ AAV capsid termed olig001, which is generated using capsid shuffling and directed evolution after rat intravenous delivery and subsequent capsid clone rescue, showing a > 95% tropism for oligodendrocytes after rat intracranial infusion.^15,16^ We hypothesize that AAVolig001 may also determine primary tropism to the SCs when delivered into the sciatic nerve.

We first generated AAVolig001-GFP transcribed by a constitutive CBA promoter, which was injected into the adult rat sciatic nerve. After injection, immunohistochemistry (IHC) revealed GFP expression selectively in both mSCs and nmSCs but not in afferent axons and somata of sensory neurons in the dorsal root ganglia (DRG). We next designed a dual promoter and bidirectional AAV construct encoding U6-driven PZ1shRNA and CBA-transcribed GFP reporter, and this construct was packed into capsid olig001 to generate AAVolig001-PZ1shRNA. *In vivo* test by intrasciatic delivery showed that AAVolig001-PZ1shRNA-mediated selective silencing of SCs-Piezo1 mitigated the development of mechanical hypersensitization while the thermal hypersensitization was spared in rats with CPNI-induced neuropathic pain. Together, our study shows that RNAi-silencing of Piezo1 via intrasciatic delivery of AAVolig001-PZ1shRNA provides a rapid and selective approach for investigating the roles of SCs-Piezo1 in peripheral nerve mechanobiology.

## Materials and methods

### Animals

Adult male Sprague Dawley (SD) rats weighing 100–125g body weight (Charles River Laboratories, Wilmington, MA) were used. Animals were housed individually in a room maintained at constant temperature (22 ± 0.5°C) and relative humidity (60 ± 15%) with an alternating 12h light-dark cycle. Animals were provided access to water and food *ad libitum* throughout the experiment, and all efforts were made to minimize suffering. All survival surgeries were completed in a sterile environment under a surgical microscope in animals anesthetized with isoflurane (2–5%). For tissue harvest euthanasia, animals were deeply anesthetized by isoflurane followed by decapitation with a well-maintained guillotine. The estimated numbers of animals needed were derived from our previous experience with similar experiments ^14,17^ and a power analysis was not performed. The numbers of rats used were detailed in the relevant sections or figure legends of the experiments.

### Molecular cloning and AAV constructs

AAV vectors encoding a dual promoter and bidirectional transgene cassette, in which a U6 promoter drives Piezo1-shRNA or a scramble RNA as control (SC) and GFP (for in vivo tracing) transcribed by a hybrid CMV enhancer/chicken β-actin (CBA) promoter, was constructed. Two shRNAs against rat Piezo1 (NM_001077200.2) were designed using Invivogen siRNA Wizard Software (https://www.invivogen.com). Piezo1-shRNA1: GCCGGCCATCTTGTTTGTTTATCAAGAGTAAACAAACAAGATGGCCGGC and 2: GCTGGAGGAGGATGACATAGATCAAGAGTCTATGTCATCCTCCTCCAGC (loop sequence underlined). The DNA sequences of U6-Piezo1-shRNAs were synthesized and subcloned into the Mlu I site by Genscript (Piscataway, NJ) that resides upstream of the CBA promoter of a single-strand AAV expressing plasmid pAAV-CBA-GFP, as we described previously.^18^ This generated pAAV-CBA-GFP-U6-Piezo1-shRNAs (pAAV-PZ1shRNAs). The plasmid of pAAV-CBA-GFP-U6-SC, designed as we previously described,^19^ was used as the control. The above plasmids were subsequently used to produce AAVs packed by capsid AAVolig001 by Packgene (Worcester, MA). A purified AAVolig001-CBA-GFP was provided by Myrtelle (https://myrtellegtx.com), and AAV6-CBA-GFP was produced and purified in our laboratory by previously established methods.^20^ Total four AAVs were used in the experiments, including 1) AAVolig001-CBA-GFP (AAVolig001-GFP, 1×10^13^GC/mL), 2) AAVolig001-PZ1shRNA-GFP (AAVolig001-PZ1shRNA, 1.2×10^13^GC/mL), 3) AAVolig001-scramble-GFP (AAVolig001-SC, 2×10^13^GC/mL), and 4) AAV6-CBA-GFP (AAV6-GFP, 2×10^13^GC/mL).

### Primary cell culture and cell lines

Primary Schwann cell cultures were performed as previously described.^14^ In brief, SCs were isolated by digesting the dissected sciatic nerve with 0.25% trypsin (Sigma-Aldrich) for a short time (10–20 s) to obtain > 80% pure SCs, as determined by immunocytochemistry (ICC) with S100, a Schwann cell marker. NG108–15 (NG108) cells were obtained from ATCC (Manassas, VA). N2A cells stably expressing CRISPR Cas9 nuclease (Cas9N2A) were obtained from Genecopoeia (Rockville, MD). These cells were cultured by a standard protocol using Dulbecco’s modified Eagle’s medium (DMED) supplemented with 10% FBS and antibiotics (ThermoFisher, Rockford, IL) and were grown at 37°C and in 5% CO2 in a humidified incubator.

### Microfluorimetric Ca ^2+^ imaging

Determination of intracellular calcium (Ca_i_^2+^) was performed using Fura2-based microfluorimetry and imaging analysis, as we previously described.^21^ Cells were imaged to monitor Ca_i_^2+^ responses to Yoda1 superfusion (1 min) and total DMSO concentration was kept equal to and below 1% for all tested Yoda1 concentrations. The Ca_i_^2+^ was measured as the ratio of emission in response to excitation at 340 and 380 nm, expressed as the 340/380 nm fluorescence emission ratio (R_340/380_) that is directly correlated to the amount of Ca_i_^2+^.^22^ A ≥ 30% increase in R_340/380_ from baseline after superfusion with Yoda1 was considered a positive response for all cells recorded.^14,23^

### AAV sciatic nerve injection

AAV was injected into the sciatic nerve using a procedure similar to that as we described for DRG injection using a microprocessor-controlled injector (Nanoliter 2000, World Precision Instruments, Sarasota, FL, USA),^24^ and comparable to a protocol described for sciatic nerve injection in mice.^25^ Briefly, after appropriate anesthesia was obtained by inhalation of 2% isoflurane, the right sciatic nerve was exposed through a lateral incision of the middle thighs and division of the superficial fascia and muscle, and the sciatic nerve was exposed at a point proximal to the bifurcation. Rats will receive sciatic nerve injection of AAV injection, in a dose of 2×10^11^ GC (~ 20μL) containing 0.1% Fast Green (0.1μL) in the viral vector solution to visualize the injected solution. AAV was injected directly into subepineural space (beneath the clear fascia surrounding the nerve but outside the perineurium) with a pulled glass capillary tip (40–60μm diameter) inserted into the sciatic nerve (~ 10mm) forming an angle with the longitudinal axis of the sciatic nerve. Once penetration was achieved, the injector was backed off until the compression of the tissue was not evident, to lessen tissue pressure on the pipette aperture. Injection was at a rate of 2μL/min over a 10-min period using a microprocessor-controlled injection system employing direct piston displacement mounted on a micromanipulator. Removal of the glass pipette was delayed for an additional 5 min to minimize the extrusion of the injectate. Following the injection and closure of overlying muscle and skin, the animals were returned to their housing where they remained as the designed experiments required. Saline (20μL) was injected as the control for the comparative evaluation of sensory behavior of injection and AAV.

### Animal pain model and behavior testing

#### Tibial nerve injury (TNI) and common peroneal nerve injury (CPNI).

Animals were anesthetized using isoflurane at 4% for induction and 2% for maintenance. Under anesthesia, the right sciatic nerve was exposed under aseptic surgical conditions by blunt dissection of the femoral biceps muscle. The sciatic nerve and its three branches (sural, common peroneal, and tibial nerves) were isolated. For TNI, the tibial nerve was then tightly ligated and transected distal to the ligation.^17^ CPNI surgery was performed using a method with minor modification, as previously validated in rats.^26^ Specifically, after exposure of the sciatic nerve and its three branches, the CPN was then tightly ligated and transected distal to the ligation (leaving the tibial and sural nerve intact). The overlying muscle and skin were then sutured following surgery. Sham-operated rats were subjected to all preceding procedures without nerve ligation or transection.

### Sensory behavioral evaluation

Behavioral tests were conducted between 9:00 AM and 1:00 PM. Animals were habituated in individual test compartments for at least one hour before each test. Behavior tests were carried out as previously described, ^24^ and were performed by personnel blind to treatments. Stimuli were applied to the hindpaw plantar skin in the tibial nerve innervating area for CPNI and in the sural nerve innervating area for TNI.

#### 1) Mild mechanical stimulation (von Frey).

The withdrawal threshold was determined using calibrated monofilaments (Patterson Medical, Bolingbrook, Illinois) with forces of 0.3, 0.5, 0.8, 1.0, 2.8, 5, 9, 14, and 24g, applied in an up-down fashion, allowing calculation of the 50% withdrawal threshold.^27^ Beginning with the 2.8g filament, filaments were applied to the plantar skin with just enough force to bend the fiber and held for 1 s. If a response was observed, the next smaller filament was applied, and if no response was observed, the next larger was applied, until a reversal occurred, defined as a withdrawal after a previous lack of withdrawal, or vice versa. Following a reversal event, four more stimulations were performed following the same pattern. The forces of the filaments before and after the reversal, and the four filaments applied following the reversal, were used to calculate the von Frey threshold. Rats not responding to any filament were assigned a score of 25g.

#### 2) Noxious mechanical stimulation (Pin).

A point of 22g spinal anesthesia needle was gently applied 5 times to the plantar surface of hindpaw with enough force to indent but not puncture the skin. Five applications were separated by at least 10s, which was repeated after 2 min, making a total of 10 touches. For each application, this evokes either a simple withdrawal response with immediate return of the foot to the cage floor or a response characterized by sustained elevation with grooming (e.g., licking or chewing the toes) and possibly shaking, lasting at least 1 s. This latter behavior was referred to as hyperalgesia behavior. This hyperalgesia response has been associated specifically with an aversive experience.^28^

#### 3) Heat stimulation.

This was performed using a device designed to identify heat sensitivity (Paw Thermal Stimulator System, University Anesthesia Research & Development Group, San Diego, CA). Rats were placed on a temperature-regulated glass platform heated to 30°C and the lateral plantar surface of hindpaw was stimulated with a radiant heat source (50W halogen bulb) directed through an aperture. The time elapsed from the initiation of the stimulus until withdrawal (withdrawal latency) as detected by a series of photocells was measured. Each hindpaw was tested four times, and the withdrawal latency values were averaged.

#### 4) Cold stimulation.

Acetone was applied from a syringe attached to PE220 tubing to make a meniscus that was touched to the plantar surface of hindpaw, such that the drop spread out on the plantar surface of the paw without contact of the tubing to the skin. Each hindpaw was tested 3 times in alternating fashion. Any withdrawal was considered a positive response. The frequency of withdrawal from the stimulus was recorded.

### Validation of Piezo1 antibody in CRISPR/Cas9-mediated Piezo1 knockout cells

Lentiviral (LV) expression plasmid pWPT-mCherry was used to express dual CRISPR guide RNAs (gRNAs) specific to mouse/rat Piezo1 (gRNA1: 5’-AGCATTGAAGCGTAACAGGG-3’, gRNA2: 5’-AGAGAGCATTGAAGCGTAAC-3’), as described previously.^14^ LVs expressing mCherry (control) or dual Piezo1 gRNAs were packaged using pWPT-mCherry and pWPT-mCherry-PZ1gRNAs with packaging plasmid pCMVDR8.74 and envelop plasmid pVSV-g, and products titrated in the range of 1×10^6^ to 1×10^7^ transduction unit/mL, as previously reported. ^14^ Cultured Cas9N2A cells grown to 50% confluence were infected by LV-mCherry-PZ1gRNAs or LV-mCherry (control) in the presence of 8μg of polybrene (Sigma-Aldrich) per mL at an optimized multiplicity of infection 5.

### Tissue harvest for immunohistochemistry (IHC) and immunoblots

The rats were ~ 4 months old when tissues were harvested. After transcardial perfusion with cold 100mL PBS, lumbar (L) 4 and 5 DRG, lumbar spinal cord, and sciatic nerve segments proximal to the sciatic bifurcation and terminal branches were dissected, and fixed in Richard-Allan Scientific^™^ Buffered Zinc Formalin (ThermoFisher) overnight, followed by processing for paraffin embedment. The previously described histological protocol was adopted.^17^

### Immunocytochemistry (ICC) and IHC

ICC on cultured cells and IHC on tissue sections were performed according to standard procedures. ^29^ Non-specific binding was reduced by incubating the sections for 30 min with a solution of 5% BSA in PBS plus 0.05% Tween20 (PBST). Cells and tissue sections were immunolabeled with the selected primary antibodies: mouse Piezo1 (1:400, Novus, NBP2–75617), rabbit GFP (1:500, Cell Signaling, CS, 2555S), rabbit mCherry (1:400, CS, 43590), rabbit GAP43 (1:400, CS, 8945), rabbit p75NTR (1:400, CS, 8238), rabbit MPZ (1:1000, CS, 57518s), rabbit S100 (1:1000, CS, 13018), goat MBP (1:1000, SCB, sc13912), rabbit NF200 (1:1000, CS, 30564), and rabbit Tubb3 (1:1000, CS, 5586), rabbit NeuN (1:400, CS, 24307s), and rabbit GFAP (1:1000, Dako, Z0334); in a humid atmosphere overnight at 4°C. The fluorophore-conjugated (Alexa 488 or Alexa 594, 1:2000) secondary antibodies (Jackson ImmunoResearch, West Grove, PA) were used to reveal immune complexes. The immunostaining was examined, and images were captured using a Nikon TE2000-S fluorescence microscope (El Segundo, CA) with filters suitable for selectively detecting the green and red fluorescence using a QuantiFire digital camera (Optronics, Ontario, NY). NIH ImageJ software (http://rsbweb.nih.gov/ij/) was used for analysis. For double-label colocalization, images from the same specimen but showing different antigen signals were overlaid by digitally merging the captured images. Positive immunostaining was defined as fluorescence intensity greater than average background fluorescence plus 2 standard deviations of the cells in an adjacent section in the same slide of negative control (the first antibody omitted) under identical acquisition parameters (n = 10 for different markers).^29^

### Immunoblots

Immunoblots of cell lysates were performed as described previously.^17^ Immunoreactive proteins were detected by Pierce enhanced chemiluminescence (ThermoFisher) on a ChemiDoc Imaging system (Bio-Rad, Hercules, CA) after incubation with HRP-conjugated second antibodies (1:5000, Bio-Rad). The densitometry of the selected bands was analyzed by NIH Image J software.

### Statistics

Statistical analysis was performed with GraphPad PRISM 9 (GraphPad Software, San Diego, CA). The numbers of biological replicates (*e.g*., animals, cells, and immunoblot samples) are provided in the corresponding figures and legends. No data points were excluded. Mechanical allodynia (vF), hyperalgesia (Pin), and thermal (heat and cold) changes after sciatic nerve injection were compared to pre-injection baseline (BL) with repeated measures of two-way ANOVA and Tukey *post hoc* for vF and heat, and Friedman’s tests and Dunn *post hoc* for Pin and cold. The area under the curves (AUC) was compared among groups by one-way ANOVA and student’s *t*-test, where appropriate. Results are reported as mean and standard deviation of the mean (SEM). Differences were considered to be significant for values at *p* < 0.05.

## Results

### AAVolig001 determines primary Schwann cell tropism after sciatic nerve delivery

Prior studies demonstrate that AAV packed by a novel oligotropic capsid olig001 shows > 95% oligodendrocyte transduction after delivery into the rat brain.^15^ We, therefore, tested whether AAVolig001 encoding GFP by a constitutive poly II promotor CBA (AAVolig001-GFP) has primary SCs tropism after intrasciatic delivery. We injected AAVolig001-GFP in a dose of 2×10^11^ GC viral particles per animal into the right sciatic nerve of adult rats. The dose was determined according to the results of a brief dose-ranging test using 5×10^10^ GC viral particles (two rats) in the pilot study for which we found less efficacy of transgene expression at a lower dose (not shown). Five weeks after injection of 2×10^11^ GC viral particles into the sciatic nerve, the animals were sacrificed, tissue harvested, and GFP expression in the ipsilateral L3-L5 DRG (and contralateral ones), sciatic nerve and its three terminal branches, and lumber spinal cord was analyzed by IHC.

**Figure 1:**
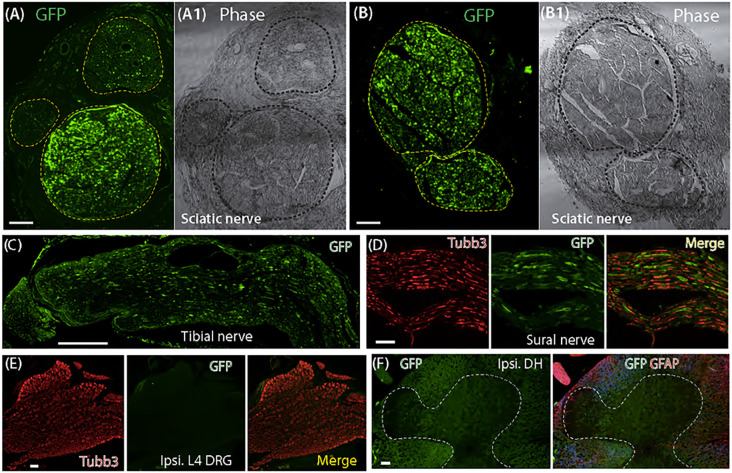
GFP expression after intrasciatic AAVolig001-CBA-GFP. Representative IHC images illustrate GFP expression in the nerve fascicles (dashed circles) of cross-sectioned sciatic nerves (A, B). Detection of GFP signals in longitudinal sections of tibial and sural serves (C, D). No GFP signals are identified in the sections for L4 DRG and spinal cord, ipsilateral to injection (E, F). Scale bar (mm): A-B, 100; C, 500; D-F, 100.

IHC revealed GFP expression in the sciatic nerves of all rats (10) injected with AAVolig001-GFP. In the sciatic nerve and its terminal branches, GFP signals exhibiting Schwann cell profile were detected in the nerve fascicles that accounted ~ 50% of the cross-sectional area of the sciatic nerve (the ages of the rats when tissues harvested were ~ 4 months old). Four out of ten rats showed higher GFP signals in the larger-diameter fascicles than in small-diameter ones and other injections showed detection of comparable GFP signals in both large and small nerve fascicles (Fig. 1A–B). GFP signals were identified in the tibial and sural nerves (Fig. 1C–D) but were low in the common peroneal nerve (not shown). In DRG and spinal cord, no apparent GFP signals were observed in sensory neurons and glia, spinal cord neurons and neuropil in dorsal and ventral horn, and spinal glial cells (Fig. 1E–F). Results suggest an *in vivo* restricted biodistribution of AAVolig001-GFP within the sciatic nerve and its branches.

**Figure 2:**
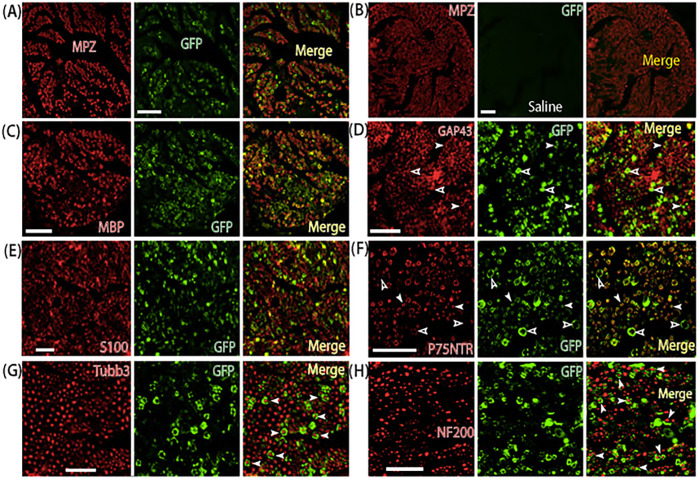
Selective Schwann cell transduction of AAVolig001-CBA-GFP. Representative IHC montage images show selective GFP expression in Schwann cells (both myelinating and non-myelinating Schwann cells), labeled by MPZ, MBP, S100, GAP43, and P75NTR (**A-F**, Panel **B** is saline injection control). Empty and white arrowheads in Panel **D** and **F** point to putative mSCs and nmSCs, respectively. No apparent GFP signals in the afferent axons labeled by Tubb3 and NF200 (**G**, **H**, arrowheads). Antibodies for double labeling are indicated in each montage IHC image. Scales: 50 mm for all. MPZ, Myelin protein zero; MBP, Myelin basic protein; S100, S100 calcium-binding protein; GAP43, Growth Associated Protein 43; P75NTR, p75 neurotrophin receptor; NF200, Neurofilament 200; Tubb3, beta3-Tubulin; Ipsi, Ipsilateral.

Co-labeling of GFP with Schwann cell makers and neuronal markers showed that GFP-positive cells exhibited the typical Schwann cell pattern, indicating AAVolig001 induction of GFP efficient and selective transduction in the SCs, but no GFP signals were found to be colocalized with the sensory neuronal markers that labeled large and small afferent fibers (Fig. 2). The cross-sectional IHC images of GFP and myelin protein zero (MPZ, a marker of nmSCs) coimmunostaining were used to quantitatively assess *in vivo* mSCs transduction since a mSC myelinates a single large diameter axon forming a single myelin sheath.^30,31^ *In vivo* transduction rate (3 rats), calculated as the GFP labeled mSCs (average 2124) out of the total MPZ positive mSCs (average 4256) in larger-diameter nerve fascicles, is ~ 50% (30 ~ 84%). AAVolig001-expressed GFP signals were also overlaid to the immunostaining of P75NTR and GAP43 that are highly expressed in nmSCs,^32–34^ indicating AAVolig001’s tropism to nmSCs. However, estimation of the transduction rate in nmSCs is difficult since a nmSC, also termed Remak Schwann cell (RSC), enfolds or accommodates multiple small-diameter non- or less-myelinated axons, forming Remak bundles.^35,36^ We, therefore, counted the percentage of P75NTR-positive small fibers that were wrapped by GFP-positive SCs, representative of estimation of AAVolig001 tropism to nmSCs. Results showed that 40 ~ 80% of P75NTR-positive small fibers were ensheathed by GFP-positive SCs. These results reveal that intrasciatic delivery of AAVolig001-GFP induces selective and efficient transduction to both mSCs and nmSCs.

**Figure 3:**
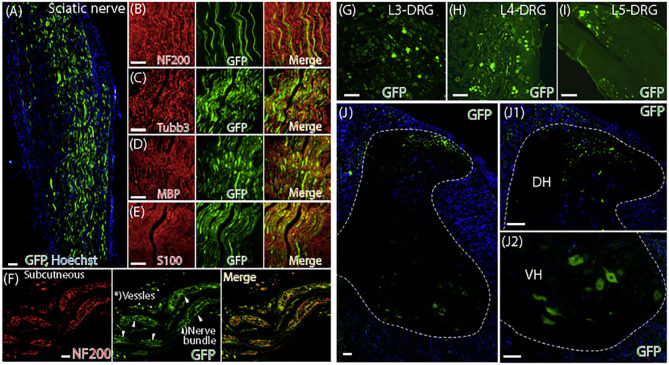
Non-cellular selective transduction after sciatic nerve delivery of AAV6-CBA-GFP. Representative IHC images illustrate that, 5 weeks after sciatic nerve injection of AAV6-CBA-GFP, GFP signals are detected in the afferent axons and SCs of the sciatic nerve by double labeling of GFP with neuronal markers (Tubb3 and NF200) and Schwann cell markers (MBP and S100) (**A-E**), as indicated. Representative IHC images show GFP signal detection, co-labeled with NF200 in the sensory terminals on the subcutaneous section **(F).** Neuronal profile of GFP signals are detected in the L3, L4, and L5 DRG (**G-I**) ipsilateral to injection. GFP signals are also detected in the spinal cord dorsal horn (DH) neuropil and ventral horn (VH) motor neurons (**J-J2**). Scale bars: 50mm for all.

For comparison, we injected AAV6-CBA-GFP in a comparable dose of 2×10^11^ GC particles (~ 20μL) into the sciatic nerve of naïve rats. IHC of GFP expression, 5 weeks after injection of AAV6-CBA-GFP, showed extensive non-cell specific GFP detection in SCs, afferent axons, sensory neuronal somata (L3-L5), neuropil in the lumbar spinal dorsal horn (SDH), and motor neurons in the spinal ventral horn (SVH) (Fig. 3). These results demonstrate that, although both AAVolig001 and AAV6 (both using the CBA promoter) efficiently transduce SCs after intrasciatic delivery, AAVolig001 has unique and selective primary tropism to the SCs in the sciatic nerve. AAVolig001 determines the SCs tropism without the need to incorporate SCs-specific elements, such as SCs-specific promoter.

### Subepineural sciatic nerve injection induces mild behavior alteration in naïve rats

**Figure 4:**
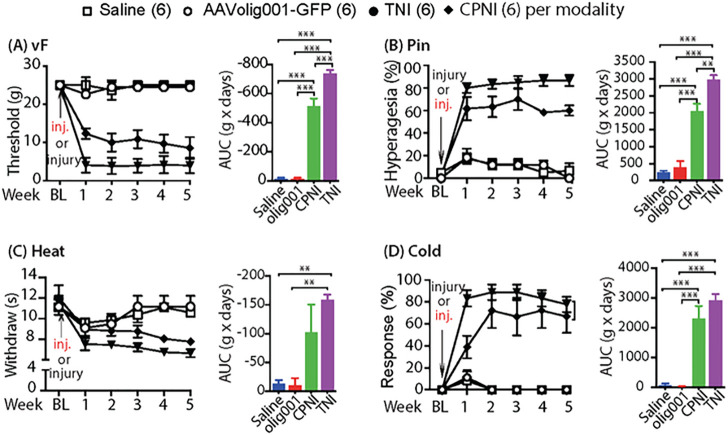
Sensory behavior valuation after intrasciatic injection of AAVolig001-CBA-GFP. The time courses for the grouped averages of sensitivity to vF (**A**), Pin (**B**), Heat (**C**), and Cold (**D**) before (baseline, BL) and after sciatic nerve injection of AAVolig001-CBA-GFP in naïve rats, compared to those of saline injection and rats subjected to TNI and CPNI (different studies in parallel with sciatic nerve injection project), as indicated (n=6 per group). Right panels of **A-D** are AUC, **p< 0.01 and ***p < 0.001, one-way ANOVA and Tukey *post-hoc*.

Sciatic nerve injection injury (SNII) has been recognized as a complication after drug delivery into the sciatic nerve.^37^ We evaluated the alterations of evoked mechanical and thermal sensory thresholds after sciatic nerve injection of AAVolig001-GFP in naïve rats, compared to sensory behaviors after saline injection in naïve rats and after peripheral nerve injury by ligation of the tibial nerve (TNI) and common peroneal nerve (CPNI). Results showed mild sensory sensitization after AAVolig001-GFP injection in naive rats, comparable to the magnitudes of minor threshold changes after saline injection, as well as to the sensitization after DRG injection of AAV to naïve rats in our previous reports;^24,38^ but the sensory alterations were significantly less than those after TNI and CPNI. The mild sensory changes after AAVolig001 injection to the sciatic nerve in naïve rats were normalized 2–3 weeks after injection, comparable to saline injection (Fig. 4). Rats after CPNI but not TNI showed no signs of movement deficiency after recovery from anesthesia.

### Cell-based knockout/knockdown validates specificity of Piezo1 antibody

**Figure 5:**
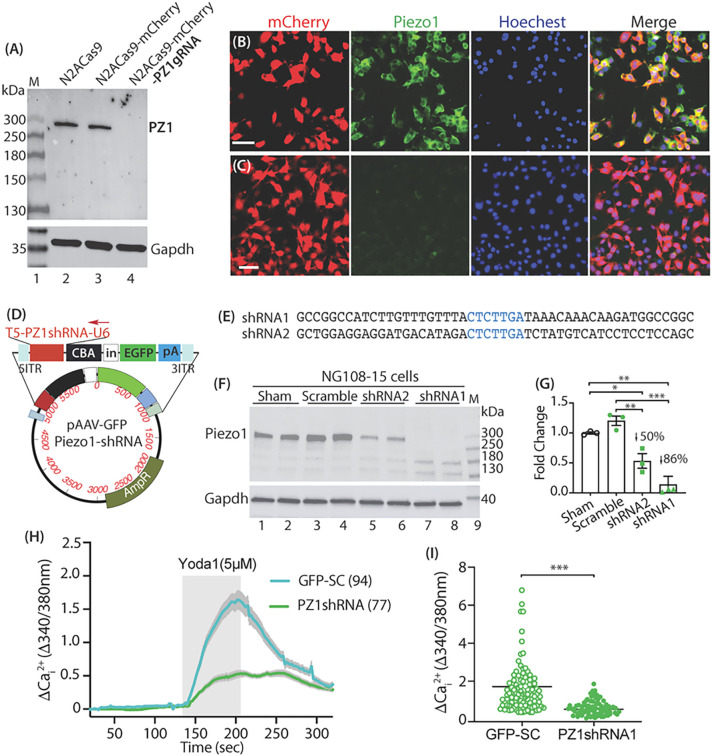
Cell-based Piezo1 CRISPR knockout and RNAi knockdown (NG108 cells). Piezo1 antibody recognizes a clean ~300kDa band of canonical Piezo1 protein by immunoblot with comparable band density in control Cas9N2A cells and Cas9N2A cells expressing mCherry, while Piezo1 band vanishes in the Cas9N2A cells expressing Piezo1-gRNA (**A**). ICC shows the detection of Piezo1 in Cas9N2A cells expressing mCherry (**B**), while Piezo1 signals are barely detected in the Cas9N2A cells expressing Piezo1-gRNA (**C**). Scale bars: 50mm for B and C. An AAV plasmid (**D**) with transgene cassette (top of the map) encoding Piezo1shRNAs (**E**) and GFP, separately, showing efficient Piezo1 knockdown after transfection of Piezo1shRNAs into NG108 cells (**F, G**). Significant reduction of Yoda1-stimulated increase of Cai2+ in NG108 cells expressing Piezo1shRNA1, compared to scramble control (**H, I**), p<0.001, two-tailed unpaired student’s t-test.

This study used a mouse Piezo1 antibody, which is an affinity chromatography purified mouse monoclonal antibody (isotope IgG2a) raised against recombinant protein encompassing amino acid 1275–1540 of human PIEZO1 (NBP2–75617, Novus Biologicals). The antibody has been validated in its specificity and has been used to detect Piezo1 expression by immunoblots, ICC, and IHC of rodent and human samples in previous studies.^39,40^ We further validated the specificity of this mouse Piezo1 monoclonal antibody to detect Piezo1 by a CRISPR-Cas9 genome editing approach using N2ACas9 cells, as described previously.^14^ Results showed that the antibody recognized a clean ~ 300kDa band predicted as the canonical Piezo1 protein by immunoblot with comparable band density in the control N2ACas9 cells and N2ACas9 cells expressing mCherry, while LV-mediated Piezo1-gRNA expression in N2ACas9 cells induced completely ablation of Piezo1 protein expression. We further validate the immunostaining specificity of Piezo1 antibody by ICC. Results showed that Piezo1 immunoreactivity vanished in the N2ACas9 cells with LV-mediated Piezo1-gRNA expression, compared to controls (Fig. 5A–C). Together, these data verify the specificity of this monoclonal antibody in the detection of Piezo1 expression by immunoblot and immunostaining.

We also designed a dual promoter and bidirectional AAV expression plasmid in which U6 promoter drives Piezo1-shRNA and CBA promoter transcribes GFP, respectively. Two Piezo1-shRNAs were constructed and tested in cell-based *in vitro* tests using NG108 neuronal cells. Results revealed that Piezo1-shRNA1 induced > 85% reduction of Piezo1 protein level after transfection into NG108 cells, compared with the sham- and scramble control (SC)-transfected cells (Fig. 5D–G). Functional Piezo1 knockdown was verified since the Yoda1-evoked increase of Cai2+ in the Piezo1-shRNA1 expressing NG108 cells was significantly reduced (Fig. 5H–I).

### Silencing Piezo1 in SCs reduces nerve injury-induced mechanosensitization

We next tested whether *in vivo* RNAi-silencing of Piezo1 selectively in the SCs will affect hindpaw hypersensitivity after peripheral nerve injury in rats. We used a common peroneal nerve injury (CPNI) model. Commonly used neuropathic pain models are ligation and transection of the tibial nerve (TNI) or tibial/sural nerves (SNI), but these two models often show both abnormal sensory and loss of motor function, and assessment of the sensory system could be affected by motor defects.^41^ The common peroneal nerve is the smaller and terminal branch of the sciatic nerve, and ligation of the common peroneal nerve was reported with long-lasting behavioral allodynia and thermal hyperalgesia but intact motor functions.^41,42^ Additionally, AAVoligo001 shows preferable biodistribution along the tibial nerve because high SCs transduction in the tibial nerve was observed more than that in common peroneal and sural nerves after AAVolig001 sciatic nerve injection. The AAVolig001 preferable tibial nerve distribution after sciatic nerve injection may induce more biological effects of SCs-Piezo1 silencing since injured terminal nociceptive territories can be re-innervated or hyper-innervated by uninjured fibers.^43–45^

**Figure 6:**
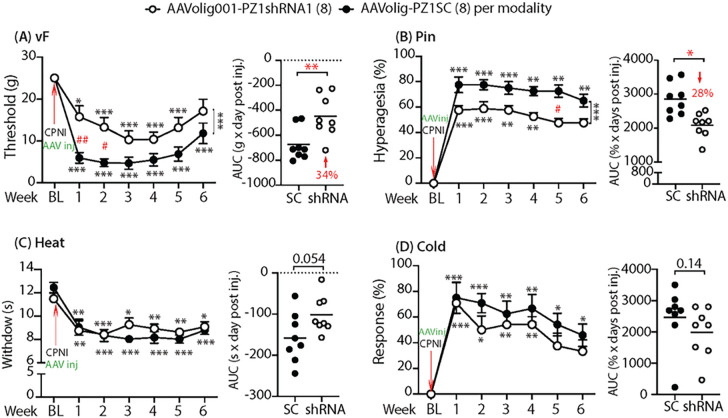
Mitigation of mechanical hypersensitivity in CPNI rats. The time courses for the grouped averages of sensitivity to vF, Pin, Heat, and Cold before (baseline, BL) and after induction CPNI immediately followed by intrasciatic injection of either AAVolig001-PZ1shRNA1 or AAVolig001-SC (**A-D**); *p<0.05, **p<0.01 and ***p<0.001 for comparisons to BL within group and ^#^*p*<0.05 and ^##^*p*<0.01 for comparisons between groups post AAV injection. Repeated measures parametric two-way ANOVA for vF and Heat and Tukey *post hoc*; and Friedman ANOVA for Pin and Cold tests and Dunn’s *post hoc*. Right panels of **A** to **D** show AUCs calculated using the measures at BL and after vector injection; *p<0.05 and **p<0.01, comparisons of AUCs between groups, unpaired and two-tailed Student’s t-test.

In the experimental design, after tests of baseline sensory behaviors, rats were randomized to two groups, AAVolig001-PZ1shRNA1 and AAVolig001-SC. Sciatic nerve injection of either vector (2×10^11^ GC viral particles) was performed immediately after CPNI surgery, and sensory behaviors were then followed on a weekly basis for 6 weeks; after that, the animals were sacrificed, and tissues were harvested for IHC determination of transgene and Piezo1 expression. Results showed that all rats in both groups developed multiple modalities of pain behaviors after CPNI surgery, including the lowered threshold for withdrawal (vF), more frequent hyperalgesic-type responses (Pin), and hypersensitivity to Heat and Acetone cold stimulation. However, hypersensitivity to the vF and Pin but not to thermal stimuli in CPNI rats subjected to AAVolig001-PZ1shRNA1 was significantly attenuated, compared to the animals injected with the control vector (Fig. 6A–D). These findings suggest that the application of AAVolig001-PZ1shRNA1 early in injury-induced neuropathic pain limits the development of hypersensitivity to mechanical allodynia and hyperalgesia.

### Validation of SCs-Piezo1 knockdown

**Figure 7:**
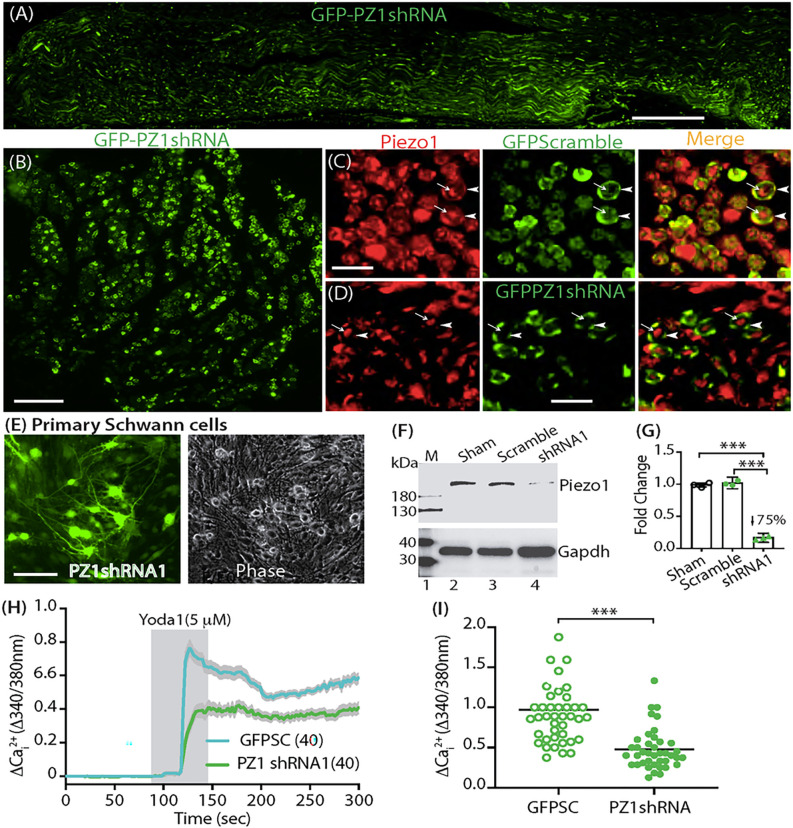
Validation of *in vivo* SCs-Piezo1 knockdown. Representative IHC images illustrate efficient GFPolig001-PZ1shRNA1 expression in the sciatic nerve after injection (**A, B**). Schwann cell (arrowheads) and axonal expression (arrows) of Piezo1 immunopositivity in AAVolig001-scramble subjected rats (**C**). SCs-Piezo1 immunostaining signals (arrowheads) are apparently reduced in AAVolig001-PZ1shRNA1 injected rats, while the axonal Piezo1 immunopositivity (arrows) was comparable to the controls (D). Immunoblots show that Piezo1 protein level in GFP-PZ1shRNA1-expressing primary cultured Schwann cells (**E**) is significantly reduced, compared to naïve and GFP control (**G, F**). Primary cultured SCs from control animals exhibit an increase of Cai2+ responding to Yoda1 stimulation, while this Yoda1-evoked response is significantly reduced in the Schwann cells expressing PZ1shRNA1 (identified by GFP signals) (**H, I**). Scale bar (mm): A, 500; B and E, 100, C and D, 10.

IHC examination on the sciatic nerve sections revealed efficient Schwann cell transduction of AAVolig001-PZ1shRNA1 6 weeks after vector injection (Fig. 7A, B). Schwann cell and axonal expression profile of Piezo1 immunopositivity in the rats subjected with AAVolig001-SC (Fig. 7C) was comparable to that as we previously described.^14^ However, the Piezo1 immunostaining signals in the SCs were apparently reduced in the rats injected with AAVolig001-PZ1shRNA1, while the axonal Piezo1 immunopositivity was comparable to the controls (Fig. 7D). This indicates that AAVolig001-PZ1shRNA1 induces selective SCs-Piezo1 silencing while axonal-Piezo1 is spared. Immunoblots of primary cultured Schwann cells showed a significant reduction of Piezo1 protein in primary cultured Schwann cells expressing PZ1shRNA1 (Fig. E-G). Primary cultured Schwann cells from control animals exhibited a robust response to the Yoda1 stimulation by an increase of Cai2+, while this Yoda1-evoked Cai2+ response was significantly reduced in the isolated Schwann cells expressing PZ1shRNA1 (identified by GFP signals) (Fig. 7H, I). Together, the data verify Piezo1 knockdown in the Schwann cells after intrasciatic delivery of AAVolig001-PZ1shRNA1.

## Discussion

Our findings show that 1) AAVolig001 has primary Schwann cell tropism via intrasciatic delivery in rats and 2) AAVolig001-PZ1shRNA-mediated selective RNAi silencing of Piezo1 in both mSCs and nmSCs alleviates the mechanical hypersensitization representing allodynia and hyperalgesia following peripheral nerve injury, while cold and heat hypersensitivity are not significantly reduced. Results indicate that SCs-Piezo1 participates in the modulation of peripheral nerve mechanosensation and that Piezo1 may function as one of the mechanotransducers in the SCs that are involved in decoding injury-induced mechanical forces into afferent nociceptive hypersensitization.^46,47^

As noted, although AAVolig001-PZ1shRNA induces selective expression and silencing of Piezo1 in both mSCs and nmSCs, the mechanical hypersensitization following CPNI is only partially reversed. This could be due to several possible reasons. AAVolig001-CBA-PZ1shRNA exhibits selective SCs tropism with an *in vivo* biodistribution restricted within the sciatic nerve after vector delivery, but the distal SCs of sensory nerve terminals are not transduced, which may limit the efficacy. In the sciatic nerve, the majority of SCs are nmSCs and most unmyelinated C-fibers ensheathed by Remak cells are nociceptors,^35^ and the sensory terminal Schwann cells are proposed to be critical in nociception.^6^ Future AAVolig001 delivery with osmatic sorbitol or mannitol may facilitate viral particle spread and transduction of terminal SCs,^48–50^ thereby enhancing analgesia. Additionally, Piezo1 is also expressed in the afferent axons that are spared transduction by AAVolig00-CBA-PZ1shRNA, and thus the total biological effects on antinociception are compromised. Furthermore, since both Piezo1 and Piezo2 are co-expressed in the SCs and Piezo2 has a proven role in nociception, partial reversal of mechanical hypersensitization by silencing of only Piezo1 may suggest that Piezo1 and Piezo2 in the Schwann cells have some functional redundancy in mechanosensation and that Piezo2 may provide functional complementary effects when Piezo1 is ablated. Future studies need to investigate the effects of AAVolig001-mediated selective silencing of SCs-Piezo2 and a combined knockdown of both SCs-Piezo1/2 on peripheral nerve mechanosensation and pain pathogenesis.

SCs are inherently mechanosensitive, and this sensitivity is important for PNS development, myelination, and regulation of sensory neurons;^51,52^ thus, gene transfer to manipulate SCs may have diverse therapeutic potential.^53^ However, a major issue for *in vivo* investigation of the roles of SCs, such as Piezo channels, in peripheral nociception is the lack of pharmacological and genetic tools that selectively target both mSCs that transmit touch and nmSCs that accommodate nociceptive nerve fibers.^54^ AAV vectors are very useful tools for selective manipulation of the genes and the associated molecular pathways for studying mechanisms and for the therapy of various PNS disorders. However, no AAV capsid has been described in the literature to exhibit primary tropism to both mSCs and nmSCs when a constitutive promoter drives transcription. Intrasciatic AAV9 with a constitutive potent CAG promoter has been shown efficient transduction to mSCs,^55^ but another study reports that intrasciatic AAV9 incorporating CMV promoter induces more widespread effects, with both SCs and other cell types transduced, including motor and sensory neurons.^56^ AAV incorporating a MPZ promoter for transcription and packed by serotype 9 has shown efficient *in vivo* transduction to SCs.^57^ However, since MPZ is known to exclusively express in the mSCs,^58^ this approach; although powerful for the investigation of mSCs; is not sufficient to study the role of nmSCs in peripheral nociception. AAV serotype 8 with a CMV promoter via direct sciatic nerve injection also transduces SCs;^59^ however, other study shows that sciatic nerve injection of AAV8 efficiently transduces sensory neurons.^60^ Our data show that, comparable to the other reports,^61,62^ sciatic nerve injection AAV6 with CBA promoter for transcription induces widespread non-cell specific transgene expression in SCs, afferent axons, sensory neuron somata, and central and peripheral terminals as well as motor neurons in the ventral horn via retrograde transduction (Fig. 3). Lentiviral vector and nanoparticles have been tested for *in vivo* mSCs transduction,^63,64^ but AAVs have superiority and are a predominant tool for *in vivo* transduction. Most current AAV capsid serotypes (using CBA or CMV promoter for transcription) via different delivery routes transduce neurons at a much higher rate than any other neural cell types, making it difficult to selectively manipulate Schwann cells.^65^ Thus true SCs-specific targeting for both mSCs and nmSCs remains elusive. AAV serotype olig001, which is a capsid with a chimeric serotype composed of AAV1, 2, 6, 8, and 9, has exclusive oligodendrocyte tropism after delivery to the brain, and in vitro tests confirm that AAVOlig001 has a greater binding affinity for oligodendrocyte surface receptors than AAV8.^15^ We verify that AAVolig001 also determines selective transduction to Schwann cells via intrasciatic delivery, without the need for Schwann cell-specific transcription elements. AAVolig001 has unique and selective primary tropism to both mSCs and nmSCs in the sciatic nerve and the transduction is predominantly localized at the site of injection, which limits potential undesirable effects in other cells and sites along the PNS that may present as confounding factors in data interpretation.

Occurrence of SNII after sciatic nerve injection may induce severe sensory disturbance and motor loss with poor recovery when delivery of toxic chemicals and the needle tips are inserted into intrafascicular. It is reported that extrafascicular space injection normally produces no or minimal nerve injury.^66^ About 50% of the cross-sectional area in the 4-month-old rats is non-neural tissue, and half of the total cross-section inside the sciatic nerve epineurium in humans consists of non-neural connective tissue.^67^ Thus, the results of behavior evaluation indicate that our injection techniques are successful in attempts to apply injecta into the extrafascicular space avoiding intrafascicular injection and that AAVolig001 itself is not toxic.^56,68^ Although it is not possible to ascertain the needle insertion sites, the minimally traumatic delivery implies that an intrafascicular injection is a preventable event in the experimental rats by our microinjection technique, which is comparable to viral vector intrasciatic injection in mice showing injection itself causing little Wallerian degeneration and long-term behavioral adverse effects on sciatic nerve functions.^25^ In comparison with genomic modification strategies such as the Cre-Loxp system, AAVolig001-based RNAi-silencing facilitates fast, reproducible, and straightforward genetic manipulation of both mSCs and nmSCs *in vivo* to investigate specific SCs molecular mechanisms in nociception, thus preventing the adaptation and compensation that frequently occur in mice with genomic modifications.^25^

## Data Availability

All experimental data generated or analyzed in this study are either included in this article or will be made available upon reasonable request.
